# Identification of High and Low-Risk Areas of Tuberculosis in Lorestan Province, West of Iran

**Published:** 2017-06

**Authors:** Jamshid Yazdani-Charati, Behzad Mahaki, Elham Ahmadi-Basiri

**Affiliations:** 1 Epidemiology & Biostatistics Department, Health Faculty, Mazandaran University of Medical Sciences, Sari, Iran,; 2 Epidemiology & Biostatistics Department, Health Faculty, Isfahan University of Medical Sciences, Isfahan, Iran

**Keywords:** Tuberculosis, Spatial pattern, Relative Risks, Lorestan province, Multilevel modeling

## Abstract

**Background:**

Nowadays, tuberculosis (TB)–an infectious disease caused by Mycobacterium tuberculosis–presents with different location patterns. Spatial analysis is one of the most important tools to detect and monitor public health disease patterns. This study aimed to identify the low and high-risk areas in Lorestan Province (west of Iran) to help the health programmer for the best intervention.

**Materials and Methods:**

Lorestan has 9 counties, 22 cities, 25 zones, 81 villages, and 2842 residential villages. Our study cases were 1481 patients registered in the TB center of Lorestan Province. We investigated the spatial distribution of TB in Lorestan between 2002 and 2008 using a multilevel model. STATA Ver. 10 software was used for the data analysis.

**Results:**

The multilevel model was a better fit to the data for the spatial correlation structure. It adjusted relative risks by borrowing information of the neighboring areas in each village. Maximum risk of disease was seen in the central zone of Koram-Abad, and all villages of Delphan were identified as low-risk areas.

**Conclusion:**

Various factors such as improvement of socio-economic conditions, implementation of programs, culture, genetic background, health-related behavior, and lifestyle can influence TB control substantially. A deprived region located in the southern part of Khoram-Abad was identified as the highest risk area in our study. The poor socio-economic structure can be an important factor for the increased risk of TB in this region.

## INTRODUCTION

Tuberculosis (TB) is the most infectious disease and caused by *Mycobacterium tuberculosis* (1). The World Health Organization declared it as a global public health emergency in 1993 (2). It is transmitted under various situations associated with lifestyles, such as shelters of homeless people, bars, and prisons (3–5). The transmission of infectious pathogens from infected to susceptible individuals diminishes by increasing the distance between them (6). Close contact between two individuals having a conversation within restricted spaces with inappropriate and insufficient ventilation is a prerequisite for being infected (3, 7).

TB causes about 1.7 million deaths in the world each year (8). There are about 9 million new TB cases annually. Its prevalence was estimated at 12 million cases by the World Health Organization in 2011 (2, 8). According to the statistical figures published by the Ministry of Health and Medical Education, the TB incidence rate is 13 per hundred thousand in Iran (9). The incidence and prevalence rate of TB is higher in the border areas of Iran, such as Sistan and Balochistan, Khorasan, Mazandaran, Gilan, West Azerbaijan, East Azerbaijan, Ardabil, Kurdistan, Khuzestan provinces, and South Beach, and these rates were low in the central parts of Iran (10, 11).

Tuberculosis, like many infectious diseases, is prone to spatial aggregation or clustering (6, 12). Previous studies of geographical patterns of tuberculosis have shown clusters of disease in the deprived areas of Madagascar, Hermosillo, Antananarivo and Mexico, a cluster surrounding a homeless shelter in an urban center in Texas, aggregation around the major urban centers in Portugal, aggregation around drinking locations in Cape Town, South Africa, and among the migrant populations in Beijing, China (1, 3, 13–16). Significant geographical variation in TB distribution has been reported across the different provinces of Iran such as Mazandaran and Ardebil. For example, the risk of the disease was lower in the rural areas compared with the urban areas of Mazandaran, and the incidence rate in the Tonekabon and Behshahr cities was higher than the mean incidence rate of the province (9, 17).

TB is associated with behavioral and demographic factors such as poor nutrition, tobacco and alcohol consumption, age, and household crowding (18, 19). Regardless of the improvements in TB control plans during the past decades, TB continues to increase globally (3).

Spatial analysis of disease prevalence and incidence has been a branch of epidemiology, public health, and the investigation of disease in human populations (20). It can be very valuable for the cost-effective intervention planning (1). Some studies of the location effects on health are based on the multilevel approach that studies geographical variations of health events by fragmenting space into arbitrary parts (21).

This study aimed to identify the high-risk areas and study the demographic patterns of this disease using an advanced statistical method consisting of a village, zone, and county. To study the spatial pattern of TB in Lorestan, we conducted a multilevel analysis at three levels. This structure takes into account the spatial autocorrelation between all the levels and smoothes the estimated risks of disease by borrowing information from neighbor regions. Lorestan has a mountainous and cold climate. The province is surrounded by Hamedan from north, Isfahan and Markazi Province from east, Kermanshah and Ilam from the west, and Khuzestan from the southern direction. It has a small border with Chahar-Mahal and Bakhtiari as well (22).

## MATERIALS AND METHODS

The presented observational ecological study is based on the notification of 1481 new tuberculosis cases (disease incidence) in villages from 2002 to 2008. Lorestan Province is located in the west of Iran between 32 degrees, 37 to 34 degrees, 22 of the north latitude from the equator and 46 degrees, 51 to 50 degrees, 30 of the east longitude from the prime meridian. According to the Population and Housing Census in 2006, the province includes 9 counties, 22 cities, 25 zones, and 81 villages (23).

Suspected patients who had TB signs and symptoms were identified based on pathological examinations and sputum smear test by private clinics or physicians of health centers and were referred to TB Registry Centers for medical treatment.

Although we have a three-level structure that we can fit in our model, it sounds better to ignore this structure and fit a simple Poisson regression model early (18). We saw that the Poisson assumption was not a good fit to the data as there is a far greater variability than would be expected from a Poisson distribution. This suggests that, in fact, the villages are not independent Poisson counts and that we should take account of some of the structure in the data by fitting random effects for the zones and counties. This will result in a Poisson response multilevel model. Standardized morbidity ratio is the response variable in this structure. The standardized mortality or morbidity ratio (SMR) is defined as the ratio of the incidence rate to the expected incidence if the incidence rates are equal to those of a reference population. The crude estimate of the SMR is obtained using the observed number of cases to the expected number of cases. When the SMR exceeds 1, there are more cases than would be expected (24). Multilevel model is a three-level model. Use of 8, 2, and 3 points at level 1, level 2, and level 3, respectively, speed up the estimation. STATA Ver. 10 software was used for the data analysis. The Akaike information criterion (AIC) was used to compare the models.

## RESULTS

Among the total cases, 58.4% were males. The mean ages of the male and female TB patients were 41.44 ± 19.18 and 42. 45 ± 21.17 yr, respectively.

First, we used the conventional Poisson regression model for spatial analysis. Then, we included random effects in the model and fit a multilevel model. The importance of the different levels of geographic aggregation was considered in the second model. The deviance was found to be 4.937 in the Poisson model. It displayed a violation of assumptions because it’s upper than 1. This most common violation is over-dispersion. We used a multilevel model to adjust for this violation. The explained variation is 0.391, 0.234 and 0.294 respectively at the village, zone and county levels. The variation between the counties is larger compared with that between the zones within the counties. The area-level variance (SE=0.080) was the lowest when the place indicators were measured across the zone spaces.

The AIC with 696.280 and 534.634 respectively in poisson regression and multilevel poisson regression models was considerably lower in the spatial multilevel model.

The multilevel model adjusted relative risks by borrowing information of the neighboring areas in each village. Smoothing was observed in areas with zero crude SMRs, in particular. As shown in table 1, the maximum risk of TB was seen in Eastern Kargaah in the central zone of Koram-Abad. Next, Western Zaz, Eastern Barirud, and urban part of Central Koram-Abad were identified as the high-risk areas, respectively.

**Table 1.** SMRs in high risk villages

**County**	**Zone**	**Village**	**SMR**	

			Crude	Smoothed
Koram-Abad	Cerntral	Eastern Kargaah	2.888	3.103
Aligudarz	Zazumahru	Western Zaz	4.003	2.554
Aligudarz	Cerntral	EasternBarirud	2.476	2.184
Koram-Abad	Cerntral	Urban part	1.890	1.869
Kuhdasht	Darb e gonbad	Darb e gonbad	1.843	1.853
Koram-Abad	DureChegeni	Dure	2.077	1.724
Aligudarz	Cerntral	Eastern Pachelak	1.736	1.499
Koram-Abad	Papi	Garit	2.622	1.492
Koram-Abad	Cerntral	Dehpir	1.385	1.492
Kuhdasht	Cerntral	Golgol	1.574	1.425

According to table 2, South Itivand, South Mirbag, and North Mirbag were identified as the low-risk areas, respectively, after Western Kakavand. In general, all villages of Delphan were identified as low-risk areas (from 0.049 in Western Kakavand to 0.138 in South Khave).

**Table 2.** SMRs in low risk villages

**County**	**Zone**	**Village**	**SMR**	

			Crude	Smoothed
Delphan	Kakavand	Western Kakavand	0.000	0.049
Delphan	Kakavand	South Itivand	0.000	0.059
Delphan	Cerntral	South Mirbag	0.000	0.069
Delphan	Cerntral	North Mirbag	0.000	0.072
Delphan	Kakavand	Eastern Kakavand	0.000	0.081
Delphan	Cerntral	North Khave	0.000	0.082
Delphan	Cerntral	Nur-Abad	0.083	0.000
Delphan	Kakavand	North Itivand	0.083	0.000
Delphan	Cerntral	Nurali	0.098	0.000
Delphan	Cerntral	South Khave	0.138	0.000
Koram-Abad	Veusian	Veusian	0.171	0.000

Smoothing effects were noticeable in areas with zero crude SMRs. Smoothing in villages such as Mahmudvand in Veisian, Chamsangar in Papi, Raazaan in Zaaghe, Azna in Central and North Beiranvand in Choghalvandi in Khoram-Abad, Pishkuh Zolgha in Besharat, and Mahru in Zazumahru in Aligudarz increased up to 0.5 unit. Most of the smoothing was seen in Mahmudvand. Its crude SMR was zero. The risk increased up to 1.18 after smoothing caused in the neighborhoods of urban parts of Central Khoram-Abad, Kakasharaf and Eastern Kaargaah in Central Khoram-Abad, Golgol in Central Kuhdasht, and Dure in Chegeni in Khoram-Abad. These areas had maximum values of crude SMR. In Mahmudvand, noticeable risk increase caused to spatial structure and correlation with such high-risk areas and borrowing information from them. According to the estimated SMRs after smoothing, the northwestern region of the province (all villages of Delphan) was identified as a low-risk region. The central part of Brujerd and Silakhor in Dorud also had a low-risk cluster in the northeast. The distribution of risks in different regions was not systematic. Except for a few low-risk clusters in the northwest and east, other explicit clusters were not identified. In general, Delphan had stable conditions in comparison with other counties. The risk of TB, according to our categorization, was low in Azna, a stable region (lower than 0.6). But the risks in the villages of Delphan were noticeably lower than those of Azna.

Selsele is located in the north and had been a jointing county with Delphan many years ago. The TB risk was low to medium in the villages of this county. Risk dispersal is the most important measure in the central part of Aligudarz as its villages belong to all the risk categories. The villages of central parts of Kuhdasht and Poldokhtar had the risk between 1 to 1.2. This can identify a cluster with medium risk and potential for higher risk in the western region of the province. Zazumahru in Aligudarz and Chegeni in Khoram-Abad contained villages with either low-risk or high-risk while western Zaz and Dure had a risk more than 1.5. Eastern Zaz and Kashkan had a risk lower than 0.6. Darb e Gonbad in Kuhdasht also had villages with the risk higher than 1.2.

## DISCUSSION

Although the incidence of TB is reducing in some advanced countries due to appropriate interventions such as health education in nutrition and identification and control of potential behaviors, the number of new cases is increasing in the developing countries because of improper nutrition-related behaviors and lack of appropriate control over some behaviors such as smoking and injecting narcotics (25). There are various factors such as culture, genetic background, health-related behavior, and lifestyle which affect tuberculosis severity among patients (26). The history of TB proves that an improvement of the socioeconomic condition and implementation of programs can influence TB control substantially. This can be achieved by promoting a healthy lifestyle, health care services, and nutrition. Economic conditions such as urbanization and lifestyle changes are associated with an increased TB risk, as it has often been in urban areas (27). Many studies showed that the risk of TB is frequently related to socioeconomic factors such as low educational status, unemployment, the number of shebeens, overcrowding, and poor housing quality (28). In a study, Waaler stated that the necessary health services for TB control should develop tremendously. However, the global fight against poverty and inequality are essential for the eradication of TB as well and should be taken into consideration (29).

Eastern Kargaah was identified as the highest risk area in our study. It is a deprived region located in the southern part of Khoram-Abad. Khoram-Abad is a mountainous area and located in a valley. Its surrounding places are surrounded by mountains with rather the same conditions, but Eastern Kargaah is located in a valley. The main livelihood is agriculture and animal farm in mountainous areas but not in the valley. This difference in geographical location and income structure can be important to form different social and economic structures and, consequently, the risk of infectious diseases such as TB. Three villages in Aligudarz were identified as high-risk areas. Western Zaz in Zazumahru was the second high-risk point. Eastern Pachelak and Eastern Barirud in the central part of Aligudarz had high risks as well. Due to the appropriate socio-economic condition in Aligudarz in the east of the province and its mountainous climate, it was not expected that several points in this area would be identified as high-risk ones. However, since most of the nomad population of the province migrate seasonally from this county and they, mostly, are smokers and addicted to narcotic drugs and, at the same time, their nutrition quality being poor, this outcome would be expected (30). Two high-risk villages were specified in the central and Darb e Gonbad zones in Kuhdasht. Kuhdasht is located near the border of Ilam and Kermanshah provinces. Marginalization is increasing in its vicinity considerably. Many rural households face economic problems, ethnic and tribal conflicts, and settle in the vicinity. Inadequate health systems may spread infectious diseases in these households which then transfer to others (31).

The villages of Delphan in the northwest of the province have the lowest risk. According to the ranking of socio-economic services, in Lorestan and its organizing based on the Scalogram model, Delphan’s ranking is 3 (30). Thus, it has appropriate socio-economic conditions compared with that of other counties. It is effective in TB control and reduction.

Due to the extensive contact of the rural population with the urbanites, urban culture, and the related requirements of it, the rural population has been exposed to social conflicts. Different studies have shown critical social challenges such as addiction and suicide in the province. Poverty and unemployment are recognized as the main causes for these problems. Mortality due to social problems (addiction, suicide, domestic violence, and homicide) shows that the longevity combined with healthy index, one of the most important indices of human development, is negative in Lorestan.

Our study showed that the empirical Bayes estimates for multilevel modeling smooth crude SMR estimates could be used to measure disease risk in small areas. This method offers a trade-off between variance reduction and bias of the estimates. In particular, when the observed cases are small, the method has been shown to produce a set of point estimates that have good properties in terms of minimizing squared error loss (32, 33). This model is an analytical method that is appropriate for data with nested sources of variability involving units at micro-units or lower level nested within units at macro-units, or higher level. It allows the investigation of both within and between group variability and prevents the inferential fallacies that usually happen when a related level is ignored (34).

With regard to the longitudinal changes and time trends in TB incidence, the effectiveness of TB control measures in special areas can be investigated longitudinally. Thus, the space-time modeling can contribute to health programming. Data collection over several years will be able to recognize spatial and temporal changes in the pattern of TB (28).

## CONCLUSION

This study describes the spatial distribution of TB across Lorestan and explores high and low-risk regions allowing for spatial autocorrelation at three levels. As efforts continue to bring TB under control in the potential areas, the spatial information can help to build efforts to target ongoing strategies, and risk factor epidemiology provides important information on who remains at risk of infection. TB in different regions continues to be ecologically associated with many of the traditional sociodemographic markers of low social and economic status. Efforts to reach high-risk low-status groups still have the potential to reap benefits.
